# Impacts of trehalose supplementation on ruminal microbiota and productivity of Japanese Black heifers under heat-stressed conditions

**DOI:** 10.5713/ab.24.0468

**Published:** 2025-01-24

**Authors:** Yasuhiro Morita, Akihisa Mukaiyama, Seiji Inoue, Kazuhisa Mukai, Shuichi Matsuyama, Satoshi Ohkura

**Affiliations:** 1 Department of Animal Sciences, Graduate School of Bioagricultural Sciences, Nagoya University, Nagoya, Japan; 2 New Business Development Unit, Food System Solutions Division, Hayashibara Co., Ltd., Okayama, Japan

**Keywords:** Cattle, Heat Stress, Microbiota, Rumen, Trehalose

## Abstract

**Objective:**

Hot environments negatively affect cattle productivity, and global warming also causes heat stress, thereby adversely impacting cattle production. Improving cattle management under such conditions is an urgent issue. Trehalose can ameliorate volatile fatty acid production and the diversity of ruminal microbiota in dairy cattle. However, no studies have evaluated on Japanese beef heifers. In this study, we investigated the effects of trehalose supplementation on the ruminal microbiota and productivity of Japanese Black heifers under heat-stressed conditions.

**Methods:**

Six cyclic Japanese Black heifers were divided into two groups: control and trehalose supplemented. The ruminal microbiota, pH, and volatile fatty acid production of these heifers were analyzed over 10 weeks in the summer in central Japan.

**Results:**

During the experimental period, the heifers in the control group showed significantly higher concentrations of ruminal acetic and propionic acids than those in the trehalose-supplemented group (p<0.05, two-way repeated measures analysis of variance [ANOVA]). The acetic/propionic acid ratio showed no significant difference between the two groups. The alpha diversity in the ruminal bacterial biota in the trehalose supplemented group was higher than that in the control group (p<0.05, two-way repeated measures ANOVA) along with a change in the beta diversity of the ruminal fungal biota (p<0.05, PERMANOVA). Linear discriminant analysis effect size analysis in ruminal microbiota identified specific microorganisms in the control and the trehalose-supplemented samples: 4 and 13 in bacteria; each one fungus; 5 and 4 protozoan families, respectively.

**Conclusion:**

Trehalose supplementation in the summer improved ruminal microbiota, especially the types of ruminal bacteria and fungi related to carbohydrate digestion, and maintained the balance of ruminal volatile fatty acid production in Japanese Black heifers. Therefore, trehalose supplementation in feed could improve cattle production under heat-stressed conditions and in global-warming scenarios.

## INTRODUCTION

Global warming also causes heat stress and the heat stress in summer negatively affects productive and reproductive performance in cattle and decreases the income of dairy and beef farmers [1,2]. A previous study indicated that production losses from heat stress were estimated to be approximately $40 billion per year by the end of the 21st century, or 9.8% of the value of meat and milk production from cattle in 2005, based on the greenhouse gas emissions (SSP5-8.5, IPCC) [3]. Under this scenario, it could amount 2.2% and 7.1% of the global milk and beef production in 2005, respectively even in 2045 [3], and the present situation could last. Therefore, improving cattle management for productivity under heat conditions is an urgent issue.

Heat stress reduces the efficiency of energy utilization owing to the higher requirements for maintenance energy [4]; therefore, cattle need to increase their energy intake. Heat stress affects dry matter intake (DMI) [5], and farmers must feed their cattle high concentrate diets to meet their energy requirements. However, decreased DMI and increased concentrate diets result in lower ruminal pH causing ruminal acidosis [6,7], which could affect ruminal microbiota and fermentation [8]. Ruminants host diverse ruminal microbiota, which are important and influence the efficiency of beef cattle production. Ruminal bacteria, archaea, viruses, fungi, and protozoa form symbiotic relationships and interactions [9]. In addition, specific ruminal microbes significantly influence ruminal fermentation in the host by reducing feed efficiency and nitrogen digestibility, and increasing methane generation [10–13]. As ruminal microbiota could play an important role in the feed efficiency of beef cattle production in hot environments and the situation of global warming, understanding them could help in improving livestock production. In addition, high environmental temperatures can cause oxidative stress in livestock, which reduces productivity through several pathways. Previous studies have indicated that oxidative stress increases in cattle under heat stress [14,15]. The relationship between general oxidative stress and gut microbiota in cow [16], pig [17], and rodents [18] were reported in the previous studies. Gu et al [16] showed the fecal microbiota was strongly associated with the degree of host oxidative stress, along with the changes in microbial composition, functions, and metabolites were different in oxidative-stressed dairy cows. Therefore, suppressing oxidative stress could be important for improving cattle production, particularly in heat-stress conditions.

Trehalose consists of two molecules of D-glucose joined by an γ,γ-1,1 glycosidic linkage, and has been reported to reduce oxidative stress in chicken [19] and dairy cows [20]. Previous studies have indicated that trehalose supplementation increases volatile fatty acid (VFA) production and ameliorates protozoan growth in dairy cows [20]. Trehalose-supplemented milk replacers help in maintaining a healthy gut environment in suckling calves [21]. However, there are no studies on the relationship between trehalose supplementation, ruminal microbiota, and production under heat-stressed conditions in beef cattle.

We hypothesized that trehalose supplementation in feed could ameliorate ruminal microbiota and improve the efficiency of ruminal fermentation in beef cattle under heat-stressed conditions. This study investigated the effects of trehalose supplementation on ruminal microbiota and productivity in Japanese Black heifers (beef cattle characterized by intramuscular fat deposition) during the summer in central Japan.

## MATERIALS AND METHODS

### Animal ethics

All experimental procedures were approved by the Committee of Care and Use of Experimental Animals of the Graduate School of Bioagricultural Sciences, Nagoya University (approval number: 2018031366).

### Animals and study site

The experiments were conducted in a farm at the Field Science Center, Graduate School of Bioagricultural Sciences, Nagoya University (35°6′ 42″ N, 137° 4′ 57″ E), Togo-town, Japan, from July to September 2020 for 10 weeks. There are four clearly distinguishable meteorological seasons in Japan; therefore, we conducted the present study during the summer (July–September). The ambient temperature and humidity in the experimental area were recorded at 1-h intervals using a data recorder (MHT-381SD; MotherTool Co., Ltd, Ueda, Japan) during the experimental period. The temperature-humidity index (THI) was calculated using the following formula:


THI=(1.8T+32)-(0.55-0.0055RH)×(1.8T-26)

where T denotes temperature in degrees Celsius and RH denotes relative humidity as a percentage [22].

Six, non-pregnant, Japanese Black heifers (*Bos taurus*, reared for beef production; aged, 11–20 months; body weight (BW), 315–376 kg) were used for this experiment. They were housed in a free stall barn with rice straw and wood dust, fed twice a day individually on mixed feed (sudan grass, timothy grass, and concentrated feed, Table 1), and provided *ad libitum* water from a water tank. The diet (BW: 350 kg, average daily gain [ADG]: 0.4 kg for Japanese Black heifer) was formulated to meet the nutritional requirements of Japanese beef heifers according to the Japanese Feeding Standard for Beef Cattle. All, non-diseased, experimental heifers underwent three additional cycles of estrus before the experiment. Moreover, all animals were raised under their respective experimental conditions for at least 1 week and were synchronized using the controlled internal drug release-synch method [23] to exclude a difference in estrous cycle.

### Experimental design

Six synchronized heifers were divided into two groups with equal age and weight distribution; one group was fed only formulated feed (control group) and the other was fed 300 g of trehalose (Hayashibara Co., Ltd., Okayama, Japan) supplement (trehalose-supplemented group) mixed in their evening formulated feed. All heifers received the feed that was formulated for this experiment two times per day (09:00h and 16:00h). Both groups of heifers were reared in the experimental stall for 11 weeks (1- and 10-week acclimation and experimental periods, respectively) under the same conditions. Ruminal fluid samples were collected, ruminal pH was recorded, and the ruminal protozoa, fungi, and microbes were analyzed. Blood samples were collected for analyzing the levels of progesterone (P4)—a marker of the estrus cycle—and thiobarbituric acid reactive substances (TBARS)—generated during oxidative stress.

### Ruminal fluid collection and ruminal volatile fatty acid and pH measurement

Ruminal fluid samples were collected non-invasively using oral stomach tubes (Ruminar; Fujihira Industry Co., Ltd., Tokyo, Japan) before morning feeding at weeks 0, 5, and 10. After ruminal fluid collection, ruminal fluid pH was measured five times, and the average value was recorded using a digital pH meter (LAQUAtwin; Horiba, Ltd., Kyoto, Japan). After pH measurement, the samples were placed in 50 mL tubes in the water bath at 38ºC, and the supernatant was filtered using a four-layer cheesecloth. The ruminal fluid samples were divided into two samples and aliquoted into 1.5 mL tubes. The divided samples were preserved at −30°C until needed for VFA measurement and ruminal microbiota analysis for the present study. Measurements of VFAs were outsourced to Mie University and conducted using a high-performance liquid chromatography system equipped with an ion-exclusion column using a post-column pH-buffered electron conductivity detection method (Shimadzu, Co., Kyoto, Japan), as described by Kondo et al [24]. The acetate/propionate molar ratio (A/P ratio) was also calculated.

### Blood sampling

Blood was collected in a heparinized tube at 14:00 h to measure plasma P4 and TBARS concentrations. Plasma was immediately separated from the blood using centrifugation (1,500×g, 30 min, 4ºC), and stored at −30ºC until needed for each blood analysis. Plasma P4 and TBARS concentrations were measured at 1- and 2-week intervals, respectively.

### DNA extraction for ruminal microbiota analysis

DNA extraction from each collected samples and dispensation of extracted DNA samples, library preparation, and identification of protozoan, fungal, and bacterial species using the 16S rRNA, TSI, and 18S rRNA gene databases of each reference strain were contracted to Bioengineering Lab. Co., Ltd. (Kanagawa, Japan). In brief, ruminal fluid samples were freeze-dried and homogenized, and the supernatants were collected for DNA extraction and divided into three samples for bacteria, fungi, and protozoa analysis. The library was prepared from the purified samples using a two-step tailed polymerase chain reaction (PCR) method. The DNA was amplified via PCR using the primers described in the next section, according to Klindworth et al [25].

### Bacterial, fungal, and protozoal biota analyses in ruminal fluid

The bacterial 16S rRNA sequence of the V3–V4 region, fungal rDNA sequence of the ITS region, and protozoan 18S rRNA sequence were amplified using two sets of primers: first-round: V3–V4f_MIX (5′-ACACTCTTTCCCTACACGACGCTCTTCCGATCT-NNNNN-CCTACGGGNGGC WGCAG-3′) and V3–V4r_MIX (5′-GTGACTGGAGTTCAGACGTGTGCTCTTCCGATCT-NNNNN-GACTACHV GGGTATCTAATCC-3′), ITS1F_KYO1(5′-ACACTCTTTCCCTACACGACGCTCTTCCGATCT-CTHGGTCATTTA GAGGAASTAA-3′), and ITS2_KYO2(5′ GTGACTGGAGTTCAGACGTGTGCTCTTCCGATCT-TTYRCTRCGTT CTTCATC-3′), and 18Sf (5′-ACACTCTTTCCCTACACGACGCTCTTCCGATCT-ATAACAGGTCTGTGATGCC-3′), and 18Sr (5′-GTGACTGGAGTTCAGACGTGTGCTCTTCCGATCT-CGGGCGGTGTGTACAAAGG-3′); and second-round: 2ndF (5′-AATGATACGGCGACCACCGAGAT CTACAC-Index2-ACACTCTTTCCCTACACGACGC-3′) and 2ndR (5′-CAAGCAGAAGACGGCATACGAGAT-Index1-GTGACTGGAGTTCAGACGTGTG-3′). Sequencing was performed using the MiSeq system with the MiSeq Reagent Kit v3 (Illumina, San Diego, CA, USA) at 2–300 bp. The Fastq_barcode_splitter from the Fastx toolkit (ver. 0.0.14) was used to selectively extract the sequences that matched the primers. Sequences with a low-quality score (<20) were removed. Pre-processed sequences were then analyzed using quantitative insights into microbiology ecology (QIIME) 2 (v2020.8) and clustered into operational taxonomic units (OTUs) based on the Greengenes database (https://greengenes.lbl.gov/Download/) for bacterial detection, UNITE (ver.8.2) for fungal detection, and SILVA (ver.128) for protozoa detection, with a 97% similarity threshold. All raw sequences were deposited in the DNA Data Bank of Japan (DDBJ; accession number: PRJDB17976).

### Plasma progesterone and thiobarbituric acid reactive substances assay

Plasma P4 concentrations were determined using a double-antibody enzyme immunoassay, as described previously by Matsuyama et al [26]. In brief, assay sensitivity was 0.1 ng/mL for 100-μL plasma samples; therefore, a sample yielding a signal below this threshold was assigned a value of 0.1 ng/mL. The intra- and inter-assay coefficients of variation were 2.8% at 5.9 ng/mL and 1.5% at 6.6 ng/mL, respectively.

Plasma TBARS concentrations were measured every 2 weeks using a TBARS Assay Kit (Cayman Chemical, MI, USA) according to Sakatani et al [15]. Standard hormones were diluted using ultra-pure water to create standard hormone dilution columns from Std 8 (50 μM) to Std 1 (0 μM). Plasma TBARS concentrations were calculated by measuring the absorbance at 540 nm using a microplate reader (iMark; BIO-RAD industries, CA, USA).

### Statistical analyses

The differences in ruminal fluid pH, values of total VFA (total value of acetic, propionic, and butyric acid) and each VFA, and A/P ratio between samples from weeks 0, 5, and 10, and the TBARS values of the 2-week-interval samples between the groups were analyzed. Two-way repeated measures analysis of variance (ANOVA) with post hoc Holm’s procedure with supplementation and sample collection timing were conducted using js-STAR XR+ release 1.7.1 (https://www.kisnet.or.jp/nappa/software/star/).

Alpha diversity metrics were calculated using the Shannon (diversity, “shannon”), Simpson (diversity, “simpson”), and Pielou (evenness) indices to detect the differences between ruminal fluid samples from weeks 0, 5, and 10 in the groups, and statistical significance among the groups was analyzed using a two-way repeated measure ANOVA with post hoc Holm’s procedure with supplementation and experimental period using js-STAR XR+ release 1.7.1 (https://www.kisnet.or.jp/nappa/software/star/). Microbiome datasets generated by high-throughput sequencing are generally compositional because they have an arbitrary total imposed by the instrument [27]. Beta diversity was assessed using principal component analysis (prcomp) of the robust Aitchison distances (vegdist, “robust.aitchison”) in all ruminal fluid samples (each of the nine samples) from each group [28]. To determine the microbiome variation attributable to individual samples, permutational ANOVA (PERMANOVA, adonis2) was performed with permutations = 100,000. These analyses were conducted using R software version 4.3.2 (http://www.R-project.org/), with the “vegan” and “stats” packages.

The linear discriminant analysis effect size (LEfSe) approach was used to identify microbial taxa that were significantly associated with groups throughout the experimental period, according to Segata et al [29]. In brief, the LEfSe algorithm contains a Kruskal-Wallis rank sum test to detect differences between classes, and linear discriminant analysis (LDA) to detect differences in the relevant features. The parameters were set at p = 0.05 and LDA score = 3.0 for computation. LEfSe was performed using the LEfSe Docker container of the biobakery account (biobakery/LEfSe v1.0.0), which was converted into the LEfSe format (format_input.py). LEfSe was executed (run_lefse.py) with specified settings and no subclass specifications.

In all statistical analysis, means were considered significant when p<0.05 and a trend when 0.05≤ p< 0.10.

## RESULTS

### Ambient conditions during the experiment and physiological changes in heifers

**Figure 1:**
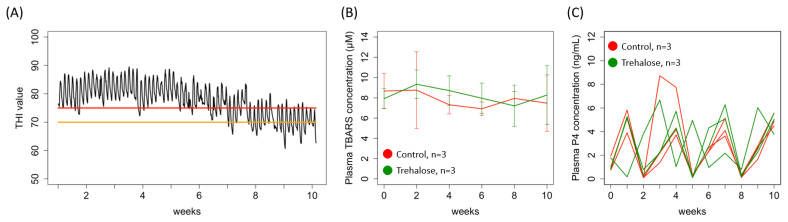
Summary of the environmental conditions during the experimental period and corresponding physiological changes in experimental heifers. (A) Average temperature-humidity index (THI) values, indicators of environmental conditions, during the experimental periods. Red and orange solid lines indicate THI values of 75 (severe heat-stressed conditions in beef cattle) and 70 (moderate heat-stressed conditions), respectively [30]. (B) Concentrations of plasma thiobarbituric acid reactive substances (TBARS) during the experimental period. These are generated during oxidative stress and are used for estimating heat stress. (C) Plasma progesterone (P4) concentrations, measured as the markers of the estrous cycle, in all heifers of both groups. Red and green solid lines indicate control and trehalose-supplemented groups, respectively.

The average THI values, which were indicators of the environmental conditions for the observed physiological response during the experimental periods, are shown in Figure 1A. On all experimental days, the maximum THI value exceeded 70. On 43 of the experimental days, the average THI value exceeded 75. Plasma TBARS concentrations during the experimental period did not differ significantly between the groups (Figure 1B). The plasma P4 concentrations in all heifers in both groups showed cyclic changes at 3-week intervals, which indicated that all heifers maintained their estrous cycles in the present study (Figure 1C). All heifers in the experiment ate the specified amounts of feed, and the gain in body weight in both groups did not differ (ADG, 0.43±0.22 vs 0.41±0.18, C vs T, mean±standard deviation [SD]).

### Changes in the volatile fatty acid concentrations and pH values in the ruminal fluid during the experimental period

**Figure 2:**
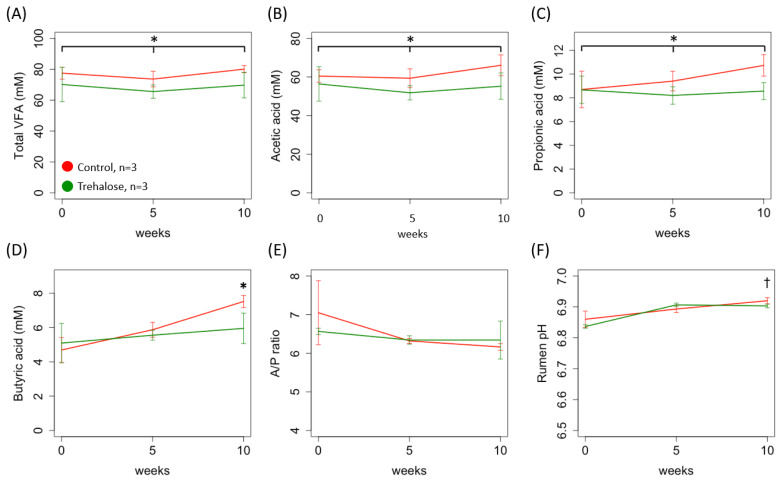
Changes in the volatile fatty acid (VFA) concentrations. (A) total VFA; (B) acetic, (C) propionic, and (D) butyric acids; (E) acetic/propionic acid ratio (A/P ratio); and (F) pH values in the ruminal fluid samples during the experimental period. Red and green solid lines indicate control and trehalose-supplemented groups, respectively. Values are means±standard deviation. * and † indicate significant difference (p<0.05 and p<0.1, two-way repeated measures ANOVA post hoc Holm’s procedure). Asterisk with solid black line in panel A and B indicates significant difference during entire experimental period (p<0.05, two-way repeated ANOVA between control and trehalose-supplemented groups).

Figure 2 shows the changes in VFA concentrations and pH values in ruminal fluid samples during the experimental period. The control group showed significantly high concentrations of total VFA, acetic, and propionic acids throughout the experimental period compared to those in the trehalose-supplemented group (77.63±7.25 vs 68.51±7.58, 61.99±5.05 vs 54.50±6.25, 9.61±1.33 vs 8.48±0.80, C vs T, mean±SD mM, p<0.05, respectively; Figures 2A–2C), and of butyric acid in week 10 (7.52±0.35 vs 5.95±0.89, p<0.05; Figgure 2D). The A/P ratio did not differ significantly between the groups (6.51± 0.59 vs 6.42±0.25; Figure 2E). The pH of the ruminal fluid at week 10 in the control group tended to be higher than that in the trehalose-supplemented group; however, this difference was small (6.92±0.01 vs 6.90±0.006, p<0.1; Figure 2F).

### Diversity of ruminal microbiota

**Figure 3:**
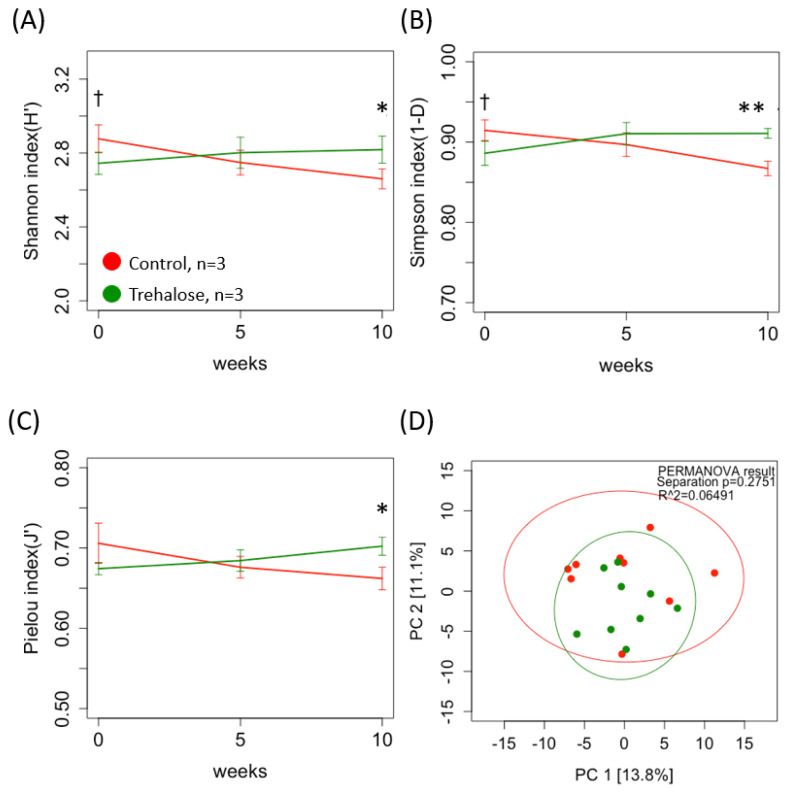
Summary of alpha and beta diversity of ruminal bacterial biota. Red and green solid lines indicate control and trehalose-supplemented groups, respectively. Alpha diversity index based on the (A) Shannon, (B) Simpson, and (C) Pielou indices. (D) Beta diversity was assessed using principal component analysis (PCA) of robust Aitchison distances within each ruminal fluid sample from each group based on the microbe 16S rRNA gene sequence data for ruminal fluid samples. Permutational multivariate ANOVA (PERMANOVA) clustering and differences in dispersion results are indicated, along with a 95% confidence interval. The red and green highlighted objects indicate specific microbes in control and trehalose-supplemented samples, respectively. Values are means±standard deviation. **, *, and † indicate significant difference (p<0.01, p<0.05, and p<0.1, two-way repeated measures ANOVA post hoc Holm’s procedure).

**Figure 4:**
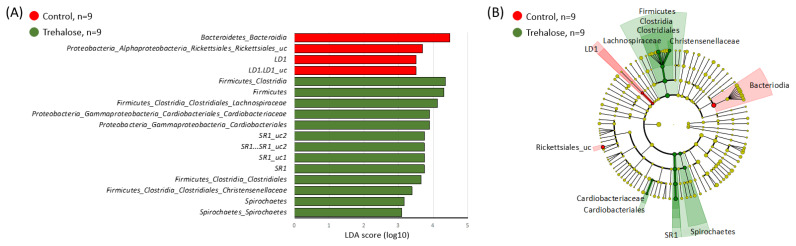
Summary of LEfSe analysis of ruminal bacterial biota. (A) Linear discriminant analysis (LDA) scores of abundant taxa using LEfSe analysis and (B) cladogram for the two groups at family level. The red and green highlighted objects indicate specific bacteria in control and trehalose-supplemented samples, respectively. LEfSe, linear discriminant analysis effect size.

Figure 3 shows the diversity of ruminal bacterial biota in both groups during the experimental period. The alpha diversity of the ruminal bacterial biota in control samples tended to be higher than that in the trehalose-supplemented samples at week 0 (2.88±0.06 vs 2.74±0.05, 0.915±0.01 vs 0.89±0.01, C vs T, p<0.1, Shannon and Simpson indices, respectively; Figures 3A, 3B). In contrast, by week 10, all parameters of alpha diversity in trehalose-supplemented samples were significantly higher than those in the control samples (2.66±0.04 vs 2.82±0.06, p<0.05, 0.87±0.01 vs 0.91± 0.005, p<0.01, 0.66± 0.01 vs 0.70±0.01, p<0.05, Shannon, Simpson, and Pielou indices, respectively; Figures 3A–3C). Beta diversity between the control and trehalose-supplemented samples showed no significant difference (PERMANOVA; p = 0.2751; Figure 3D). LEfSe analysis revealed 17 bacteria associated with the control (n = 4) and trehalose-supplemented samples (n = 13). *LD1* spp. and *Rickettsiales* spp. were specifically identified in the control samples, whereas *SR1*, *Lachnospiraceae, Cardiobacteriaceae, Christensenellaceae*, and *Spirochaetes spp* in the trehalose-supplemented samples (Figures 4A, 4B).

**Figure 5:**
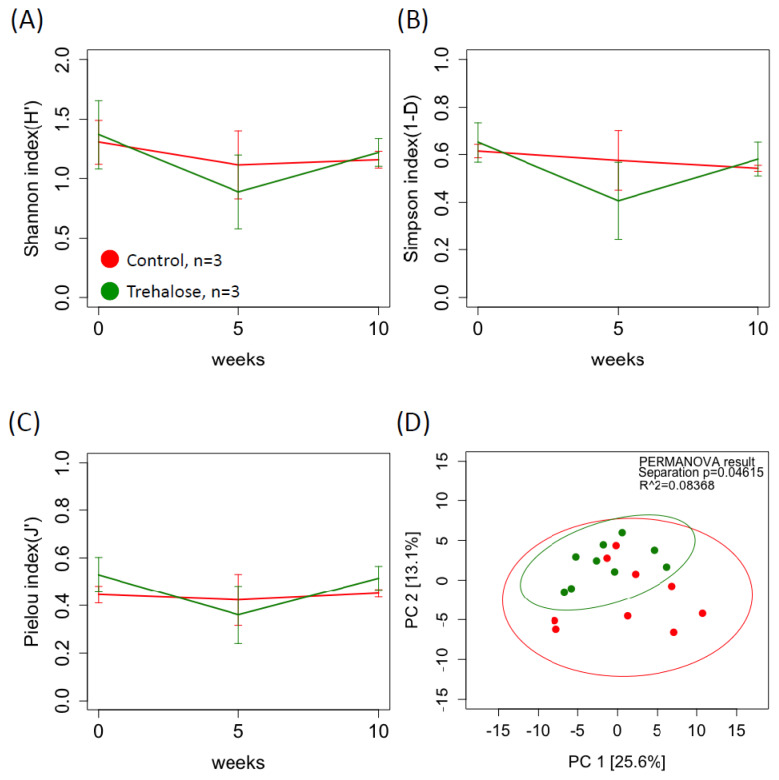
Summary of alpha and beta diversity of rumen fungal biota. Alpha diversity index based on the (A) Shannon, (B) Simpson, and (C) Pielou indices. (D) Beta diversity was assessed using principal component analysis (PCA) of robust Aitchison distances within each ruminal fluid sample from each group based on the fungi ITS rRNA gene sequence data for ruminal fluid samples. Red and green solid lines indicate control and trehalose-supplemented groups, respectively. Permutational multivariate ANOVA (PERMANOVA) clustering and differences in dispersion results are indicated, along with a 95% confidence interval. Values are means±standard deviation.

**Figure 6:**
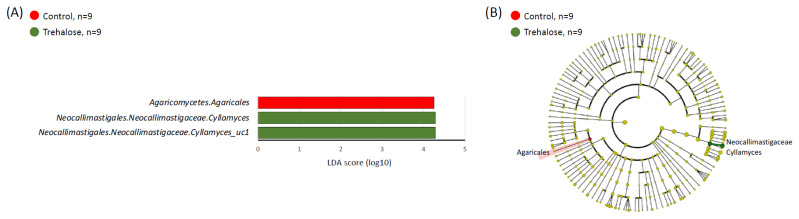
Summary of LEfSe analysis of ruminal fungal biota. (A) Linear discriminant analysis (LDA) scores of abundant taxa using LEfSe analysis and (B) cladogram for the two groups at strain levels. The red and green highlighted objects indicate specific fungi in control and trehalose-supplemented samples, respectively. LEfSe, linear discriminant analysis effect size.

Figure 5 shows the diversity of the ruminal fungal biota in both groups during the experimental period. In the relative abundance of the ruminal fungal biota, the *Neocallimastigales* family was relatively most abundant in all samples (97.96±2.13%), and therefore, fungal biota was analyzed at the species level. There were no significant differences in the alpha diversity indices (Figures 5A–5C) between the two groups. In contrast, there was a significant difference in beta diversity between the two groups (PERMANOVA; p = 0.04615; Figure 5D). In the LEfSe analysis, the fungi strains, *Agaricales spp*. and *Cyllamyces spp*., were specifically identified associated with the control and trehalose-supplemented samples, respectively (Figures 6A, 6B).

**Figure 7:**
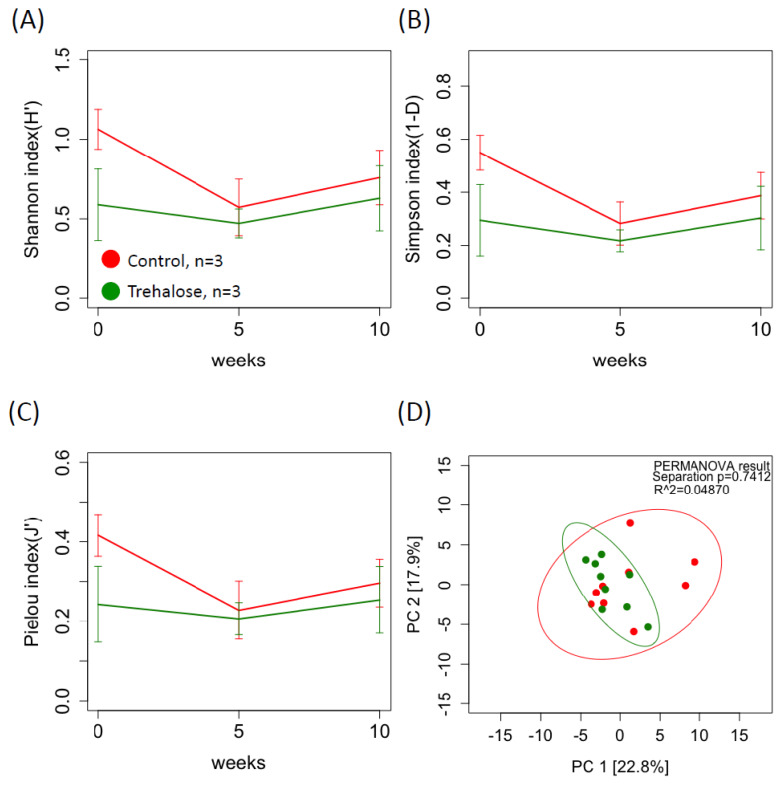
Summary of alpha and beta diversity of the rumen protozoal biota. Alpha diversity index based on the (A) Shannon, (B) Simpson, and (C) Pielou indices. (D) Beta diversity assessed using principal component analysis (PCA) of robust Aitchison distances within each ruminal fluid sample from each group based on the protozoal 18S rRNA gene sequence data for ruminal fluid samples. Red and green solid lines indicate control and trehalose-supplemented groups, respectively. Permutational multivariate ANOVA (PERMANOVA) clustering and differences in dispersion results are indicated, along with a 95% confidence interval. Values are means±standard deviation.

**Figure 8:**
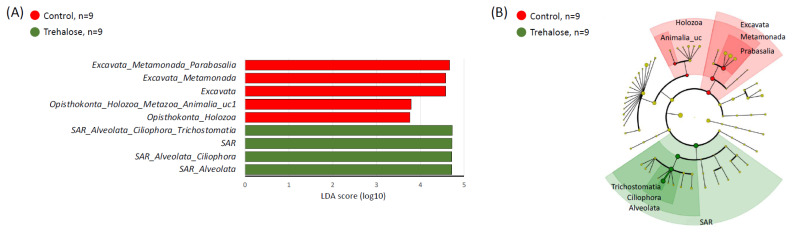
Summary of LEfSe analysis of ruminal protozoal biota. (A) Linear discriminant analysis (LDA) scores of abundant taxa by LEfSe analysis and (B) cladogram of the two groups at family level. The red and green highlighted objects indicate specific protozoa in control and trehalose-supplemented samples, respectively. LEfSe, linear discriminant analysis effect size.

Figure 7 shows the diversity of ruminal protozoan biota in both groups during the experimental period. There were no significant differences in the alpha diversity indices (Figures 7A–7C) of both groups, and there was no clear trend in beta diversity (PERMANOVA; p = 0.7412; Figure 7D). In contrast, LEfSe analysis revealed five protozoan families associated with the control and four associated with the trehalose-supplemented group. Unclassified *Parabasalia* and *Animalia* were specifically identified associated with the control samples, whereas *Trichostomatia*, and unclassified *Ciliophora* and *Alveolata* were specifically identified associated with trehalose supplementation (Figures 8A, 8B).

## DISCUSSION

Trehalose supplementation in feed during the summer period increased alpha diversity in ruminal bacterial biota by changing the beta diversity in fungal composition, whereas there were no significant changes in the A/P ratio of ruminal VFA. During the experimental period, the maximum THI value of each day exceeded 70, and on 43 of the experimental days, the average THI value exceeded 75, which is classified as a moderate and severe heat-stress condition in beef cattle respectively [30]. On the other hand, no heifer experienced oxidative stress during the experimental period. TBARS value is one of the parameters of oxidative stress. However, previous report has suggested its inadequacy as an indicator of oxidative stress, such as the lack of correlation between the TBARS value and antioxidants, such as sulfhydryl groups, in the blood [31]. In the present study, we could not confirm the efficiency of trehalose supplementation in improving the oxidative stress response in Japanese Black heifers under heat-stressed condition.

On the relationships between trehalose supplementation and ruminal VFA production in the present study, total VFA production seemed to be decreased in the trehalose-supplemented samples. However, in the present study, total VFA did not involve minor VFA and the trehalose-supplemented samples might contain these minor VFA. Therefore, trehalose supplementation could influence VFA composition diversity. In the present study, the butyric acid content in ruminal samples was significantly higher in the control group than in the trehalose-supplemented samples. A previous study indicated that butyric acid production increases during subacute ruminal acidosis [32]. The higher propionate and valerate concentrations in ruminal VFA could be also the high-risk factors for ruminal acidosis [33]. Therefore, the control group had a higher risk of ruminal acidosis than the trehalose-supplemented group. In addition, Trehalose supplementation could increase alpha diversity in the ruminal bacterial biota and prevent ruminal acidosis. Maintaining and improving alpha diversity in ruminal bacterial biota could ameliorate ruminal fermentation. Alpha diversity in the ruminal bacterial biota may be essential for avoiding ruminal acidosis and maintaining ruminal VFA production [8,34]. Moreover, the risk of ruminal acidosis could be increased in hot environments due to heat stress, which affects DMI and VFA metabolism in the rumen [35,36]. Therefore, trehalose supplementation could improve cattle production under heat-stressed condition.

Microbes in the Firmicutes phyla were specifically identified in the trehalose-supplemented samples in the LEfSe analysis, and the Firmicutes/Bacteroidetes ratio (F/B ratio) increased along with supplementation. An increase in the F/B ratio is related to obesity in humans and animals [37,38]. However, the metabolic characteristics of obesity may be desirable in beef cattle, because cattle production requires efficient fat management [39].

The unclassified family of *LD1* was specifically found in control samples by LEfSe analysis. The abundance of *LD1* was lower in the ruminal fluid of high-yield dairy cattle with high starch-containing feed [40] and higher in cattle raised on grass [41]; therefore, the abundance of *LD1* in the ruminal fluid could be related to the efficiency of a grass-based diet. In contrast, *Lachnospiraceae*, *Christensenellaceae*, and *Cardiobacteriaceae*, which are minor families in the rumen, were detected in trehalose-supplemented samples by LEfSe analysis. A previous study indicated a relationship between *Lachnospiraceae* abundance and carbohydrate metabolism in the rumen and that microbes have a highly adaptive metabolic potential and strong plasticity in response to progressive increases in dietary starch [42]. *Christensenellaceae* are generally detected in the mammalian intestine and rumen [43], and may be involved in carbohydrate digestion in the rumen of Japanese Black cattle [44]. *Lachnospiraceae* and *Christensenellaceae* could be related to changes in ruminal fermentation during the fattening period in Japanese Black cattle raised on diets with high starch content [44,45]. Therefore, trehalose supplementation may affect carbohydrate metabolism in the rumen and improve cattle growth. In addition, the alpha diversity in the bacterial community was changed after a 10-week trehalose supplementation. The ruminal bacterial and fungal microbiota analysis with trehalose supplementation in cattle is the first time, and there were no studies for these community. The previous study only indicated trehalose supplementation for 3 days affected protozoa composition in the rumen of dairy cows [20]. On the other hand, another study that trehalose supplementation for 55 days affected fecal microbiota more compared with the supplementation for 22 days in calves [21]. Therefore, trehalose supplementation may need around two months to affect ruminal microbiota, particularly bacterial and fungal communities. Therefore, trehalose supplementation should be conducted more than two months before the summer season.

Trehalose supplementation changed the beta diversity in ruminal fungal composition, indicating that trehalose supplementation could support and/or suppress specific fungal families. In the present study, LEfSe analysis revealed that *Agaricales* were specifically identified in the control group. *Neocallimastigales* were highly abundant in both groups in the present study. *Neocallimastigales* are anaerobic ruminal fungi that are important for the breakdown of complex cellulose-rich materials during fermentation in the rumen [46]. *Cyllamyces*, which are anaerobic genera with bulbous morphotypes, are specifically found in trehalose-supplemented group in the present study, and also frequently found in fresh cattle fecal samples [47]. No studies have been conducted on the relationship between *Cyllamyces* activity and trehalose supplementation. Although further study is needed, trehalose could also change ruminal fungal composition as well as ruminal bacterial diversity changes.

Trehalose did not affect alpha and beta diversity of the ruminal protozoal biota in the present study. These results are inconsistent with those of a previous study in which trehalose supplementation ameliorated ruminal protozoan growth in dairy cows [20]. This difference might be caused by the sampling procedure and ruminal characteristics of the hosts. In the present study, we collected ruminal fluid orally and removed residues from the samples; therefore, protozoa may have been caught during the filtering procedure. In addition, protozoan abundance in the rumen may differ between dairy and beef cattle because the ruminal environment may be related to feed characteristics [48,49] and age [50].

In conclusion, although there are limitations that the sample number was small and the effect of long-term trehalose supplementation were not considered in the present study, trehalose supplementation during the hot season could alter ruminal bacterial biota and support the balance of ruminal VFAs production. Therefore, trehalose can be used as feed supplementation to improve cattle productivity under heat-stressed conditions, and farmers could prevent their production and income loss. The cattle production could be maintained and increased to meet future demands as well as under global warming scenarios.

## Figures and Tables

**Table 1:** Summary of feed for experimental Japanese black heifers

	CP (g)	TDN (kg)	DM (kg)	Ca	P	CF%
Nutrient requirement for experiment heifers^1)^	635	3.88	6.64			
Nutrient composition of feed	657	3.60	6.25			
Nutrient adequacy of feed	1.04	0.93	0.94	0.29	0.25	29.1

1) BW, 350 kg, DG, 0.4 kg/day.

CP, crude protein, TDN, total digestible nutrient, DM, dry matter, Ca, calcium, P, phosphorus, CF%, crude fiber ratio; BW, body weight; DG, daily gain.
